# Cystatin C proteoforms in chronic kidney disease

**DOI:** 10.1371/journal.pone.0269436

**Published:** 2023-02-01

**Authors:** Helene Dahl, Klaus Meyer, Kristina Sandnes, Natasha Lervaag Welland, Iselin Arnesen, Hans-Peter Marti, Jutta Dierkes, Vegard Lysne

**Affiliations:** 1 Department of Clinical Medicine, Centre for Nutrition, University of Bergen, Bergen, Norway; 2 Bevital AS, Bergen, Norway; 3 Department of Clinical Medicine, University of Bergen, Bergen, Norway; 4 Department of Nephrology, Haukeland University Hospital, Bergen, Norway; 5 Department of Medical Biochemistry and Pharmacology, Haukeland University Hospital, Bergen, Norway; 6 Mohn Nutrition Research Laboratory, University of Bergen, Bergen, Norway; University of Health Sciences Gulhane Faculty of Pharmacy, TURKEY

## Abstract

Cystatin C, a cysteine protease inhibitor, is used as a biomarker of renal function. It offers several advantages compared to creatinine, and formulas for the estimation of the glomerular filtration rate based on cystatin C have been developed. Recently, several proteoforms of cystatin C have been discovered, including an intact protein with a hydroxylated proline at the N-terminus, and N-terminal truncated forms. There is little knowledge about the biological significance of these proteoforms. **Methods**: Cross-sectional study of patients with different stages of chronic renal disease (pre-dialysis n = 53; hemodialysis n = 51, renal transplant n = 53). Measurement of cystatin C proteoforms by MALDI-TOF MS, assessment of medicine prescription using the first two levels of the Anatomical Therapeutic chemical system from patients’ records. **Results**: Patients receiving hemodialysis had the highest cystatin C concentrations, followed by pre-dialysis patients and patients with a renal transplant. In all groups, the most common proteoforms were native cystatin C and CysC 3Pro-OH while the truncated forms made up 28%. The distribution of the different proteoforms was largely independent of renal function and total cystatin C. However, the use of corticosteroids (ATC-L02) and immunosuppressants (ATC-H04) considerably impacted the distribution of proteoforms. **Conclusion**: The different proteoforms of cystatin C increased proportionally with total cystatin C in patients with chronic kidney disease. Prescription of corticosteroids and immunosuppressants had a significant effect on the distribution of proteoforms. The biological significance of these proteoforms remains to be determined.

## Introduction

Chronic kidney disease (CKD) is affecting about 11–13% of the general population and its prevalence increases with age [1]. It is defined as “abnormalities of kidney structure or function, present for at least three months, with implications of health” [2]. Usually, the estimated glomerular filtration rate (eGFR) is used for the classification of CKD into different stages, from the least severe stage 1 to the most severe stage 5. At stage 5, also called end-stage renal disease (ESRD), the renal function is diminished to a point where survival in most patients becomes dependent on renal replacement therapy (RRT). RRT consists of hemo- or peritoneal dialysis or renal transplantation. Renal transplantation is the treatment of choice, with its enhanced patient survival, better quality of life and lower costs compared to dialysis treatment [3].

eGFR is usually estimated by equations including age, sex, and race, in addition to measures of serum creatinine [4]. The use of serum creatinine as a marker of renal function has been criticized, as muscle mass and dietary intake also influence serum levels of creatinine [5]. In recent years, formulas for eGFR based on cystatin C (CysC) or both CysC and creatinine have been developed and tested [6, 7]. It has been shown that CysC based formulas of eGFR can improve the risk prediction associated with kidney function [8].

CysC is a cysteine protease inhibitor that belongs to the type 2 cystatin gene family, encoded by the CST3 gene. The physiological role of cystatins is to regulate the activity of endogenous proteinases, which are often secreted or leaked from the lysosomes of dying or diseased cells. CysC was discovered in 1961, first in cerebrospinal fluid and the complete amino acid sequence was determined in 1981. It is non-glycosylated and consists of 120 amino acids and has a molecular mass of 13,343 Da. At the N-terminus, CysC has a conserved glycine at position 11 that is common to all cystatins [9]. The N-terminal segment of cystatin is of great importance for both binding affinity and inhibition specificity. Truncation of human CysC by 10 peptides at the N-terminal reduces the inhibition of cathepsin B and cathepsin H by more than 1,000-fold and 50-fold, respectively [10, 11].

All nucleated cells produce CysC at a constant rate and the protein is freely filtered in the renal glomeruli with no re-absorption. The proximal tubular cells in the kidney are also the main catabolic site of CysC. The protein is almost completely cleared from the circulation by glomerular ultrafiltration. In urine, the content of CysC is negligible in physiological conditions but raises due to damage of proximal tubular cells [9]. Due to the free filtration, CysC may reflect renal function more precisely than serum creatinine, especially in mildly decreased renal function, and several studies have shown that use of CysC-based equations for estimating GFR resembles closer to measured GFR than creatinine- based equations [7, 9]. Additionally, CysC is less influenced by muscle mass and dietary intake than serum creatinine [12]. Several drugs, however, have been described to affect CysC production, among these are corticosteroids, often used in patients who have received a renal transplant [13].

Protein biomarkers and protein heterogeneity have attracted attention during the past years in the purpose of diagnostics, risk assessment, and therapy for diseases. Post-translational modifications (PTMs) of proteins, resulting in proteoforms is one of the processes that increase the protein heterogeneity that may vary between both individuals and diseases. A better understanding of PTMs may be key to personalized diagnostics, treatment, and prognostics [14]. In recent years, more than 12 different proteoforms of CysC have been identified [15, 16], and five of these are commonly detected in plasma or serum, including three N-truncated proteoforms and two prolyl- hydroxylated proteoforms [17–20]. However, it is unclear at present whether these proteoforms increase proportionally with the severity of CKD, and whether they may affect the use of CysC as a marker of kidney disease. It was therefore the aim of the current study to measure proteoforms of CysC in patients with various stages of CKD and to relate these proteoforms to disease severity, mode of treatment, and eGFR.

## Materials and methods

A cross-sectional, single-center observational study was carried out at Haukeland University Hospital in Bergen, Norway. The study was conducted in accordance with principles of the Declaration of Helsinki and was approved by the Reginal Committee for Medical and Health Research Ethics at the University of Bergen (REK Vest, No. 2014/1790).

Adult patients at different treatment stages of CKD were included in the study; pre-dialytic CKD-patients stage 3–5 (CKD 3–5), ESRD patients receiving hemodialysis (HD), and patients after renal transplantation (KTX). Patients were recruited from November 2014 until July 2018. To be included the patients had to be 18 years or older, speak and understand English or Norwegian, be able to give informed consent, and should be clinically stable, and in case of HD patients, in steady state. Written informed consent was obtained from all patients.

Information about medical history and lifestyle factors was obtained from the patients by questionnaires. Further, anthropometric measurements were conducted by trained personnel. A non-fasting blood sample was obtained, in patients receiving hemodialysis pre-dialysis and after the long interval, and in the other patients at the outpatient clinic. Additional information, especially on prescribed medicines, was retrieved from the patient’s electronic medical record. Prescribed medicines were grouped according to the Anatomical Therapeutic Chemical (ATC) classification system using the first and second level [21]. Laboratory analysis of routine variables and the eGFR was performed by the Central Laboratory at Haukeland University hospital, using the CKD-EPI equation based on creatinine measurements. In addition, we applied eGFR calculations based on equations including CysC or both CysC and creatinine [6]. Serum CysC and its proteoforms were quantified by immuno-MALDI-TOF MS by Bevital AS [19]. Briefly, CysC antibodies were immobilized onto C18 reversed-phase tips (Ziptips, Millipore; Billerica, MA), washed intensely with Phosphate buffered saline and water, and the antigen was finally eluted with 7ul of 1% Trifluoroacetic acid. The sample was mixed with 5ul of 2,5- dihydroxyacetonephenone matrix and the mixture was analyzed by MALDI-TOF MS. CysC was quantified using a poly-histidine tagged recombinant variant of CysC as internal standard.

The following forms were analyzed:

Total CysC: sum of all proteoforms

CysC native: unmodified CysC

CysC 3Pro-OH: 3-proline hydroxylated CysC

CysC des-S: N-terminal serine truncated CysC

CysC des-S 3Pro-OH: N-terminal truncated serine and 3-proline hydroxylated CysC

CysC des-SSP: N-terminal serine-serine-proline truncated CysC

We present both absolute concentrations of the proteoforms and their proportion of total CysC, to correct for increasing total CysC concentrations at lower renal function.

### Statistical analysis

Descriptive statistics including the CysC proteoforms are presented for all patients, and for each patient group (CKD 3–5, HD, KTX) separately. We calculated proportions of proteoforms of total CysC, to take into account the compositional nature of these data, and to be able to look at distributions in addition to the absolute concentrations.

We calculated whether differences in eGFR based on formulas using either creatinine or CysC differed across the range of eGFR (Bland-Altman plot) and whether these differences were related to the different proteoforms by linear regression and Pearson correlation analysis.

To evaluate the compositional change in the CysC proteoforms across different levels of eGFR (based on creatinine) as an indicator for disease status, we performed a Dirichlet regression adjusted for patient group, use of medications from the ATC classes H02 (corticosteroids) and L04 (immunosuppressants), and C-reactive protein (CRP) as a marker for inflammation. Patients with ESRD were excluded from these analyses. The predicted proportions from the model were plotted as a function of decreasing eGFR, superimposed on the observed proportions. Additionally, each proteoform was considered separately by beta regression analyses to identify associations of the proportion of proteoforms with eGFR (as an indicator for disease status), patients’ group, medications, and CRP.

We did additional beta regression analyses for different medications, according to the first two levels of the ATC classification for those drug classes used by at least 10% of the patients [21].

All statistical analyses were made using R version 4.03 (R Foundation for Statistical Computing, Vienna, Austria), and the packages within the *Tidyverse*, *DirichletReg*, *betareg*, and *blandr*.

## Results

### 1. Characteristics

In total, 157 patients were included in the study, of which 53 (34%) patients were CKD 3–5 patients, 51 (32%) HD patients, and 53 (34%) KTX patients. The majority of the patients were male (72%) and the age ranged from 21–89 years. The lowest total CysC-values were observed in KTX patients, and the highest in HD patients. The characteristics and the concentration of the different CysC proteoforms per patients’ group are presented in Table 1, per stage of estimated GFR in S1 Table.

**Table 1:** Characteristics of the study population.

Variable	Total	CKD 3–5	HD	KTX
N	157	53	51	53
Age, years	58.9 (16.3)	59.6 (16.5)	59.9 (18.3)	57.3 (14.1)
Female patients, n (%)	44 (28.0)	15 (28.3)	12 (23.5)	17 (32.1)
BMI, kg/m^2^	26.1 (4.8)	28.1 (5.5)	24.1 (3.9)	25.9 (4)
Prescribed medicines, n (%)				
ATC-H02	65 (41.4)	5 (9.4)	9 (17.6)	51 (96.2)
ATC-L04	63 (40.1)	3 (5.7)	7 (13.7)	53 (100.0)
eGFR, mL/min/1.73m^2^				
Creatinine	30.4 (24)	31.5 (12.2)	6.25 (1.78)	52.6 (22.3)
Creatinine & CysC	26.5 (21.4)	27.4 (12)	6.61 (1.64)	44.8 (22.3)
CysC	25.2 (19.9)	25.7 (12.8)	8.72 (3.19)	40.5 (22.4)
Creatinine, μmol/L	375 (318)	216 (117)	775 (221)	144 (70)
Urea, mmol/L	16.6 (7.8)	15.4 (6)	22.8 (6.6)	11.9 (6.3)
CRP, mg/L	5.0 (12.9)	3.4 (3.9)	8.8 (21.2)	3.0 (3.7)
Total CysC, mg/L	3.49 (2.16)	2.68 (1.1)	5.63 (1.7)	2.26 (1.82)
CysC native	1.19 (0.72)	0.89 (0.37)	1.84 (0.56)	0.87 (0.7)
CysC 3Pro-OH	1.41 (0.93)	0.99 (0.41)	2.36 (0.73)	0.92 (0.79)
CysC des-S	0.26 (0.17)	0.23 (0.1)	0.42 (0.18)	0.14 (0.1)
CysC des-S 3Pro-OH	0.38 (0.24)	0.34 (0.14)	0.61 (0.22)	0.2 (0.14)
CysC des-SSP	0.25 (0.17)	0.22 (0.1)	0.4 (0.16)	0.14 (0.11)

Continuous variables presented as means (SD) and categorical variables reported as counts (%). eGFR based on equations for creatinine and/or cystatin C by CKD-EPI [6].

BMI, body mass index; CKD 3–5, pre-dialysis chronic kidney disease stage 3–5; CysC, Cystatin C; CysC native, unmodified CysC; CysC 3, Pro-OH: 3-proline hydroxylated CysC; CysC des-S, N-terminal serine truncated CysC; CysC des-S 3Pro-OH, N-terminal truncated serine and 3-proline hydroxylated CysC; CysC des-SSP, N-terminal serine-serine-proline truncated CysC; eGFR, estimated glomerular filtration rate; HD, end-stage renal disease hemodialysis; KTX, renal transplant recipient. ATC-H02 –corticosteroids, ATC-L04—immunosuppressants

### 2. Association of patients’ group, prescribed medicines and eGFR with proportion of CysC proteoforms

#### Patients’ group

Native CysC and CysC 3Pro-OH were the major proteoforms in all patients’ groups, followed by the truncated proteoforms. The proportion of the proteoforms for all patients and for each treatment group and the explained variance by treatment group by Beta regression is shown in Table 2. Further adjustment for age, sex, BMI, CRP and eGFR did not substantially change the explained variance of the native CysC-forms and increased the explained variance of the truncated forms from 28% to 35% for CysC des-S, from 62% to 65% for CysC des-S 3Pro-OH and from 15% to 22% for CysC des-SSP. While the variance of the proportion of native CysC was explained to a large extent (72% in the unadjusted model and 42% for CysC 3Pro-OH, respectively), the variance in the proportion of the truncated forms was explained to a much lower extent (28% of the variance for CysC des-S, 62% for CysC des-S 3Pro-OH, and 15% for CysC des-SSP).

**Table 2:** Beta regression analysis of the proportion of CysC proteoforms among patients’ groups. The table shows the proportion (%, 95% CI) of total CysC and the explained variance by patients’ group (R^2^).

Proteoform %	CKD 3–5	HD	KTX	p	R^2^
CysC native	33.4 (32.9, 33.8)	32.8 (32.1, 33.4)	38.6 (37.9, 39.2)	<0.001	0.72
CysC 3Pro-OH	37.1 (36.5, 37.7)	42.0 (41.0, 42.9)	40.2 (39.3, 41.1)	<0.001	0.42
CysC des-S	8.9 (8.4, 9.3)	7.3 (6.7, 7.9)	6.5 (6.0, 7.1)	<0.001	0.28
CysC des-S 3Pro-OH	12.6 (12.2, 12.9)	10.8 (10.4, 11.2)	9.0 (8.6, 9.3)	<0.001	0.62
CysC des-SSP	8.4 (7.8, 9.0)	6.9 (6.2, 7.7)	5.8 (5.2, 6.5)	<0.001	0.15

CysC, Cystatin C; CysC native, unmodified CysC; CysC 3, Pro-OH: 3-proline hydroxylated CysC; CysC des-S, N-terminal serine truncated CysC; CysC des-S 3Pro-OH, N-terminal truncated serine and 3-proline hydroxylated CysC; CysC des-SSP, N-terminal serine-serine-proline truncated CysC; CKD 3–5, pre-dialysis chronic kidney disease stage 3–5, HD, end-stage renal disease hemodialysis; KTX, renal transplant recipients

#### Prescribed medicines

We investigated further whether prescribed medicines were associated with the differences in the proportion of the different proteoforms. The frequency of prescription of medicines belonging to the different ATC first level differed among the groups (S2 Table). Table 3a shows the effect of drugs belonging to ATC-classification (first level) on the explained variance by a certain drug group and the change in the proportion of the proteoforms in those patients with such a medicine (unadjusted values). Table 3b shows the effect of drugs (ATC classification 2^nd^ level, only those with at least one prescription from this level, on the explained variance and the change in the proportion of the proteoforms (unadjusted values).

**Table 3:** Beta regression on the association of prescribed drugs with the proportion of CysC proteoforms.

**A**						
**ATC group**	**No. of patients taking at least one drug of this group**	**Native CysC**	**CysC 3Pro-OH**	**CysC des-S**	**CysC des-S 3Pro-OH**	**CysC des-SSP**
**A: Alimentary tract and metabolism**	139	**0.01** -0.8	**0.05** +2.1	**0** -0.3	**0.01** -0.5	**0.01** -1.0
**B: Blood and blood forming organs**	107	**0.09** -2.0	**0.06** +1.6	**0.01** +0.3	**0.01** +0.3	**0.01** -0.7
**C: Cardiovascular system**	145	**0** -0.6	**0** +0.1	**0.01** +0.7	**0** +0.1	**0** -0.7
**G: Genito-urinary system and sex hormones**	18	**0** -0.1	**0** +0.3	**0** +0.1	**0** -0.1	**0.01** -0.6
**H: Systemic hormonal preparations**	84	**0.32** +3.5	**0.05** +1.4	**0.17** -1.5	**0.33** -2.2	**0.04** -1.1
**L: Antineoplastic and immunomodulating agents**	65	**0.51** +4.4	**0.03** +1.0	**0.15** -1.4	**0.41** -2.4	**0.08** -1.5
**M: Musculo-skeletal system)**	47	**0.01** +0.7	**0** -0.3	**0** +0.1	**0** -0.3	**0.01** -0.4
**N: Nervous system**	50	**0.03** -1.1	**0.02** +1.0	**0** +0.0	**0** +0.3	**0** -0.1
**R: Respiratory system**	17	**0** -0.3	**0** +0.3	**0** -0.3	**0** -0.0	**0.01** +0.8
**V: Various**	51	**0.21** -3.0	**0.17** +2.7	**0.01** -0.3	**0.01** +0.3	**0** +0.4
**B**						
**ATC group**	**No of patients with at least one prescription**	**Native CysC**	**CysC 3Pro-OH**	**CysC des-S**	**CysC des-S 3Pro-OH**	**CysC des-SSP**
**A10: Drugs used in diabetes**	22	**0.02** -1.2	**0** +0.2	**0.13** +1.8	**0.02** +0.7	**0.17** **-2.5**
**A11: Vitamins**	85	**0.19** -2.7	**0.02** +1.0	**0** +0.2	**0.03** +0.7	**0.01** +0.6
**A12: Mineral supplements**	74	**0.07** +1.6	**0.12** +2.1	**0.07** -1.6	**0.19** -1.6	**0.06** -1.3
**B01: Antithrombotic agents**	98	**0.05** -1.4	**0.06** +1.6	**0** +0.2	**0** +0.1	**0.03** -0.9
**B03: Antianemic preparations**	58	**0.18** -2.7	**0.16** +2.6	**0** +0.0	**0.01** +0.3	**0** +0.2
**C03: Diuretics**	54	**0.06** -1.5	**0.03** +1.0	**0.01** +0.4	**0.01** +0.4	**0.01** -0.6
**C09: Agents acting on the RAA system**	88	**0** -0.3	**0.04** -1.3	**0.06** +0.9	**0.03** +0.6	**0** -0.0
**C10: Lipid-modifying agents**	98	**0** +0.3	**0.03** +1.0	**0** +0.2	**0.02** -0.6	**0.05** -1.3
**H02: Corticosteroids**	65	**0.54** +4.6	**0.02** +0.8	**0.12** -1.3	**0.41** -2.4	**0.09** -1.6
L04: Immunosuppressants*	63	**0.56** +4.7	**0.02** +0.9	**0.16** -1.5	**0.42** -2.4	**0.09** -1.6
**N05: Psycholeptics**	37	**0.05** -1.6	**0** +0.4	**0** +0.3	**0.03** +0.8	**0.01** +0.6
**V03: Various**	51	**0.21** +2.7	**0.17** +2.7	**0.01** -0.3	**0.01** +0.3	**0** +0,4

**A**:

Each cell provides the explained variance (R^2^) by ATC classification (first level, in bold) and the mean difference (in %) between drug users and non-users for the unadjusted model.

CysC, Cystatin C; CysC native, unmodified CysC; CysC 3, Pro-OH: 3-proline hydroxylated CysC; CysC des-S, N-terminal serine truncated CysC; CysC des-S 3Pro-OH, N-terminal truncated serine and 3-proline hydroxylated CysC; CysC des-SSP, N-terminal serine-serine-proline truncated CysC;

**B**:

Each cell provides the explained variance (R^2^) by ATC classification (second level, in bold) and the mean difference between drug users and non-users for the unadjusted model. Only ATC subgroups that have been prescribed to at least 10% of patients (n = 16) and that had significant associations (p<0.05) with at least 2 proportions are presented. The full overview on prescribed drugs is presented as S2 Table)

* *n = 40 on mycophenolate mofetil, n = 21 on tacrolimus, n = 27 on cyclosporin.

CysC, Cystatin C; CysC native, unmodified CysC; CysC 3, Pro-OH: 3-proline hydroxylated CysC; CysC des-S, N-terminal serine truncated CysC; CysC des-S 3Pro-OH, N-terminal truncated serine and 3-proline hydroxylated CysC; CysC des-SSP, N-terminal serine-serine-proline truncated CysC;

#### eGFR

We further explored the association of eGFR (based on creatinine) with the proportion of the different proteoforms by Dirichlet regression in 102 patients with complete data on all covariates, not including patients receiving hemodialysis. Results are shown in Fig 4. In general, a decrease in eGFR was associated with a reduced proportion of the native and the hydroxylated (3Pro-OH) proteoforms and increased proportions of the truncated forms (des-S, des-S 3Prd-OH, and des-SSP). Beta-regression of the individual proteoforms demonstrated the same associations and suggested that the effect was primarily driven by the differences in medication between the patient groups (S3 Table). While a reduction of eGFR was strongly associated with % changes in the proportion of proteoforms in the unadjusted model, this association disappeared for all forms except CysC des-SSP after adjustment in all models. Prescription of drugs was different among the patients’ groups (Table 1, S2 Table).

### 3. Association of proteoforms with differences in eGFR in CKD and TX patients

**Fig 1:**
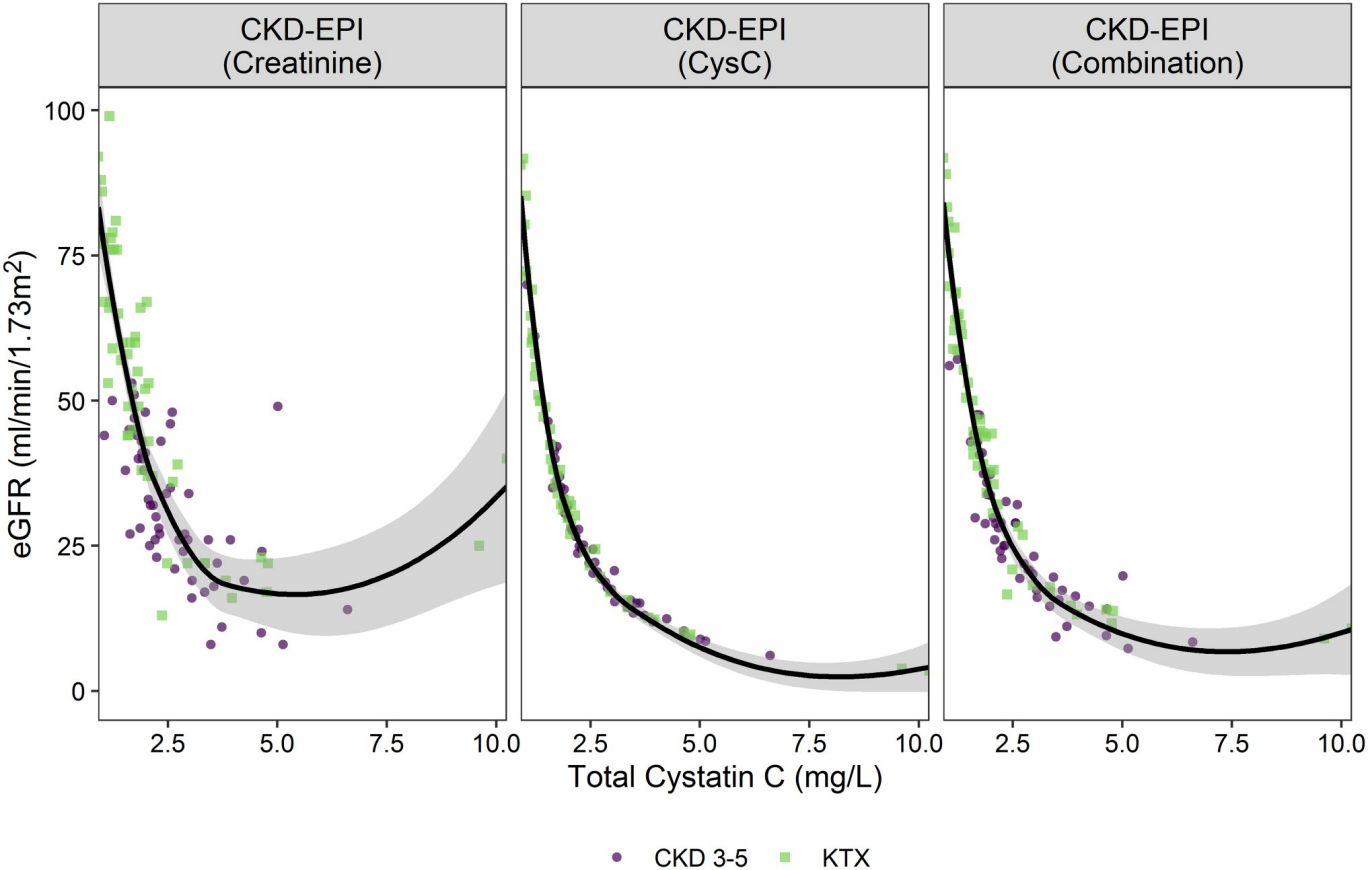
The association between total cystatin C and eGFR calculated based on creatinine, cystatin C, or both in CKD patients (CKD 3–5 and transplant). The individual data points are colored according to patient group, and the overall association is superimposed as a smoothed spline (95% CI).

The association of total CysC with eGFR in both CKD and TX patients is shown in Fig 1. Patients receiving hemodialysis were left out since there are uncertainties associated with the calculation of eGFR in this patient group. Fig 1 shows the association with eGFR based on the CKD-EPI equation based on creatinine, CysC, and the combination of CysC and creatinine, respectively.

**Fig 2:**
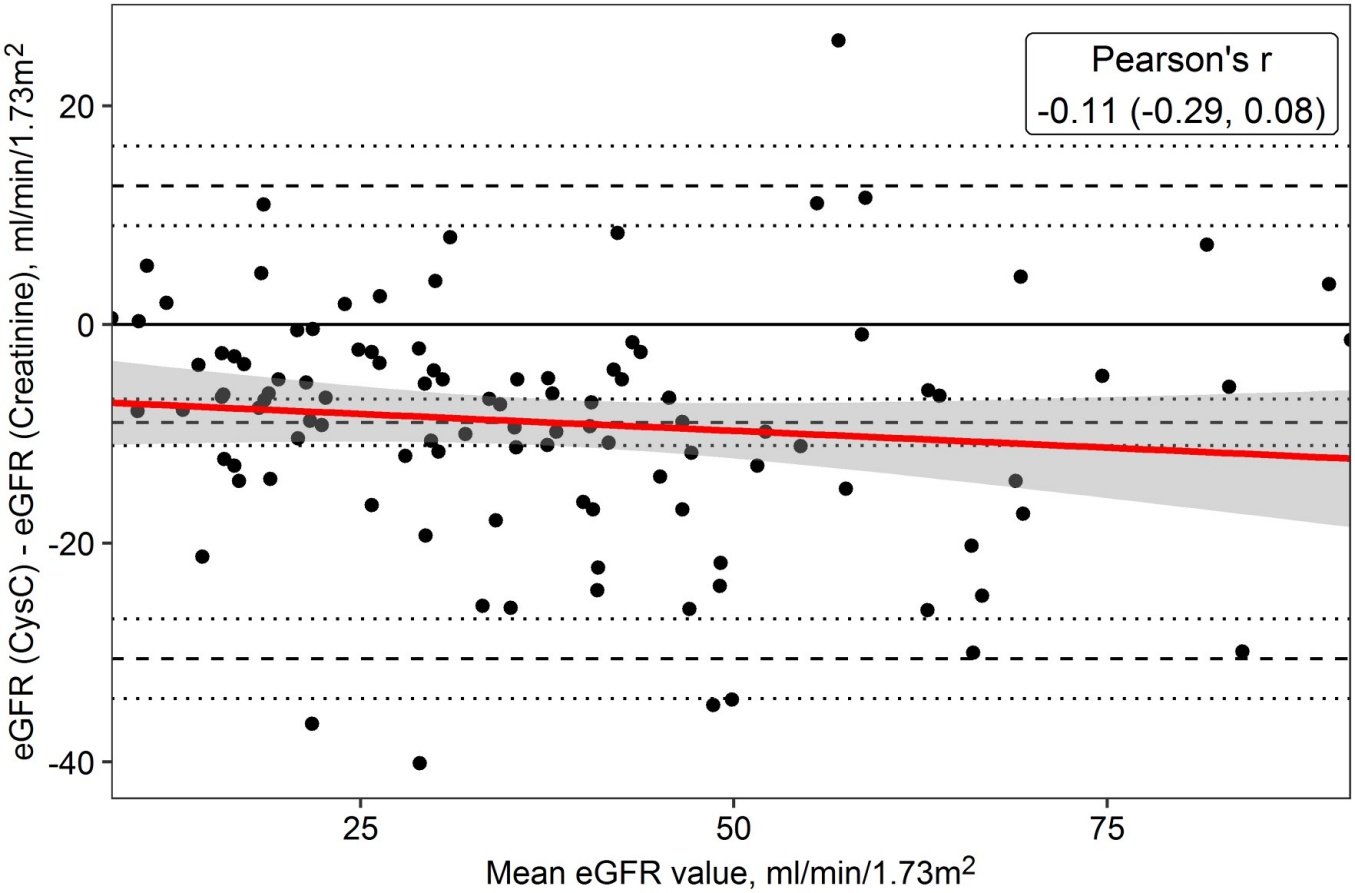
Bland-Altman plot on the differences between eGFR_cysc_ and eGFR_crea_ in CKD patients (CKD 3–5 and transplant). The superimposed regression line indicates the association between the differences and the mean value.

We further explored whether the difference between the eGFR_cysc_ and eGFR_crea_ was associated with the different proteoforms. First, we estimated whether the difference between eGFR_cysc_ and eGFR_crea_ and was associated with the magnitude of eGFR (Bland-Altman-plot). The mean difference was -9.0 ml/min (eGFR_cysc_ was lower), with a 95% confidence interval of -6.8 to -11.1 ml/min. The disagreement between the methods was slightly larger for higher eGFR values (Pearson’s r -0.11, p = 0.27, Fig 2).

**Fig 3:**
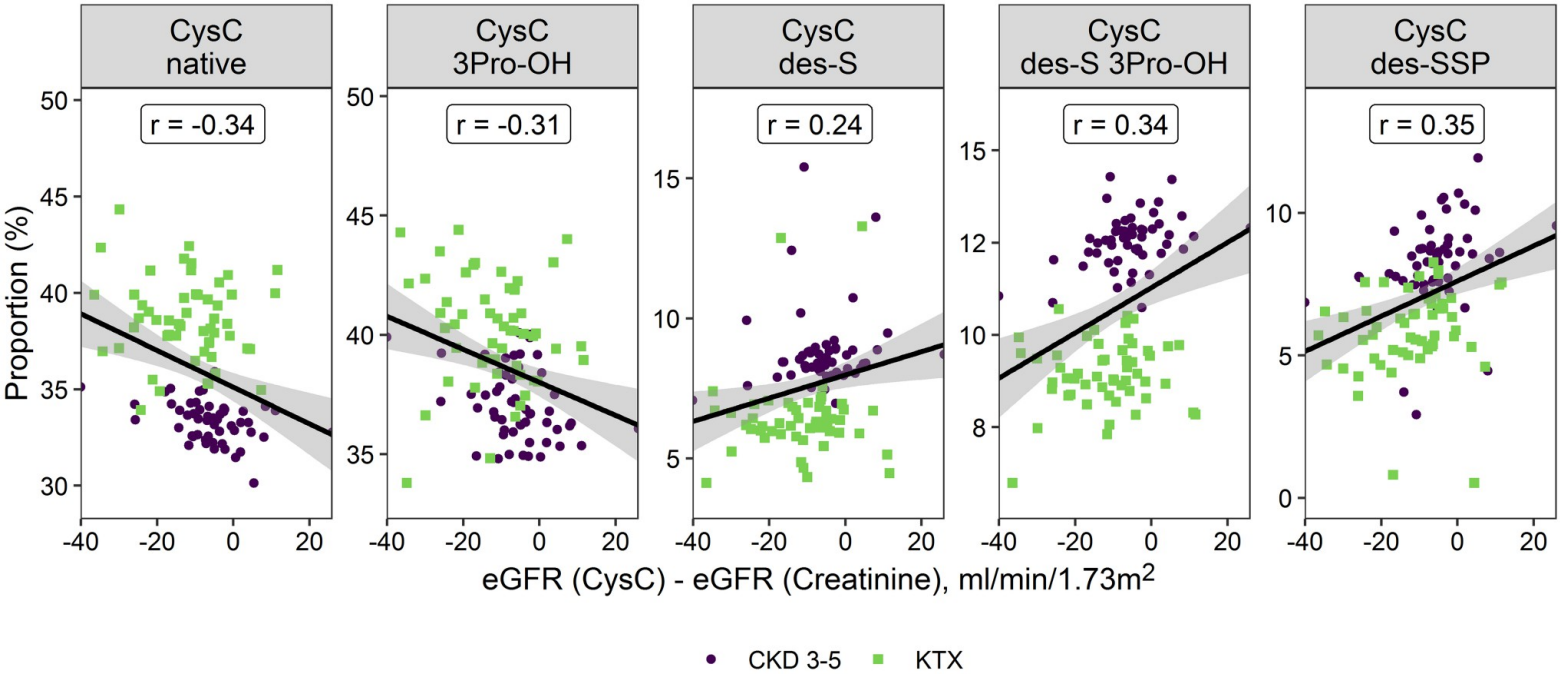
Pearson correlation between the proportion of the different proteoforms and the difference in eGFR based on creatinine and cystatin C (eGFR_cysc_–eGFR_crea_) in CKD patients (CKD 3–5 and transplant). The individual data points are colored according to patient group.

The differences between the two formulas (eGFR_cysc_—eGFR_crea_) were associated with the proportion of the different proteoforms, with a positive correlation between the (mostly negative) difference and the truncated proteoforms (indicating that the more the eGFR_cysc_ deviated from eGFR_crea_, the lower the proportion of truncated proteoforms) and a negative correlation between the difference and the native proteoforms (Fig 3).

## Discussion

In this study, we have measured total CysC and its proteoforms in patients at different stages of CKD, including patients with mild CKD, and end-stage renal disease receiving hemodialysis and after receiving a renal transplant. The total CysC concentration is increasing by decreasing renal function. Also, the absolute concentrations of the different proteoforms are increasing at decreasing renal function, in line with earlier findings [17, 18]. However, even though eGFR was associated with the proportion of the different proteoforms in the unadjusted models, other factors as patients’ group and prescription of corticosteroids and immunosuppressants had stronger effects which became apparent after adjustment. These factors have not been investigated so far and may question the clinical importance of the different proteoforms of CysC.

In line with earlier findings, native CysC and CysC 3Pro-OH were the most abundant proteoforms in the patients, and both forms contributed to about 70–80% of total CysC. All patients had measurable N-terminal truncated CysC proteoforms (CysC des-S and des-SSP) and CysC-des-S that contained a hydroxylated proline residue at the N-terminus. The differences in the proportion of these forms among the groups was not explained by eGFR but differed according to prescribed medicines. This has not been shown before.

The biological significance of the observed proteoforms is unclear at present. Both prolyl hydroxylation and N-terminal truncations posttranslational modifications and may have important regulatory functions and implications. Prolyl hydroxylation in collagen, for example, is required for collagen maturation and formation of stable collagen fibrils [22]. In the hypoxia-inducible factor (HIF), prolyl hydroxylation is related to the stability of the protein [23]. However, even if the amino acid sequence in CysC is compatible with prolyl hydroxylation by prolyl-4-hydroxylase which acts on proline residues on growing and newly synthesized polypeptide chains [22], there is hardly any information on the consequences of prolyl hydroxylation of CysC. Truncations at the N-terminal site of CysC have revealed that the N-terminus is critical for the binding of cystatins to the target, and that there is a highly conserved glycine residue at position 11. Removal of the N-terminus before or after this residue leads to a marked reduction in the affinity for target proteins [24], but these studies investigated longer truncations than the 3 amino acid residues that lack in the CysC des-SSP. In fact, the effect of the removal of these three amino acids on CysC activity or binding has not been investigated at all.

We investigated whether different proportions of proteoforms are associated with differences in estimated GFR, calculated with formulas based on either creatinine or CysC. It is known that CysC-based equations may predict true GFR better than creatinine-based equations, especially in mild chronic renal disease [6]. Thus, the rationale behind this was that posttranslational modifications may either affect the measurement of CysC or association of CysC with CKD. It has to be taken into account that in the present study, cystatin C was measured by immuno-MALDI-TOF MS, as opposed to nephelometric methods which are used in clinical routine [19]. However, good agreement of the immune-MALDI-TOF assay with the nephelometric methods has been shown earlier [19, 20]. Indeed, we observed strong correlations of both the native and the truncated forms with the difference between eGFR_cysc_ and eGFR_crea_. Higher proportion of the truncated forms were associated with lower differences in the eGFR, while higher proportion of the native forms were associated with larger differences in the eGFR (compare Fig 3). However, there was no obvious underlying explanation for these associations.

**Fig 4:**
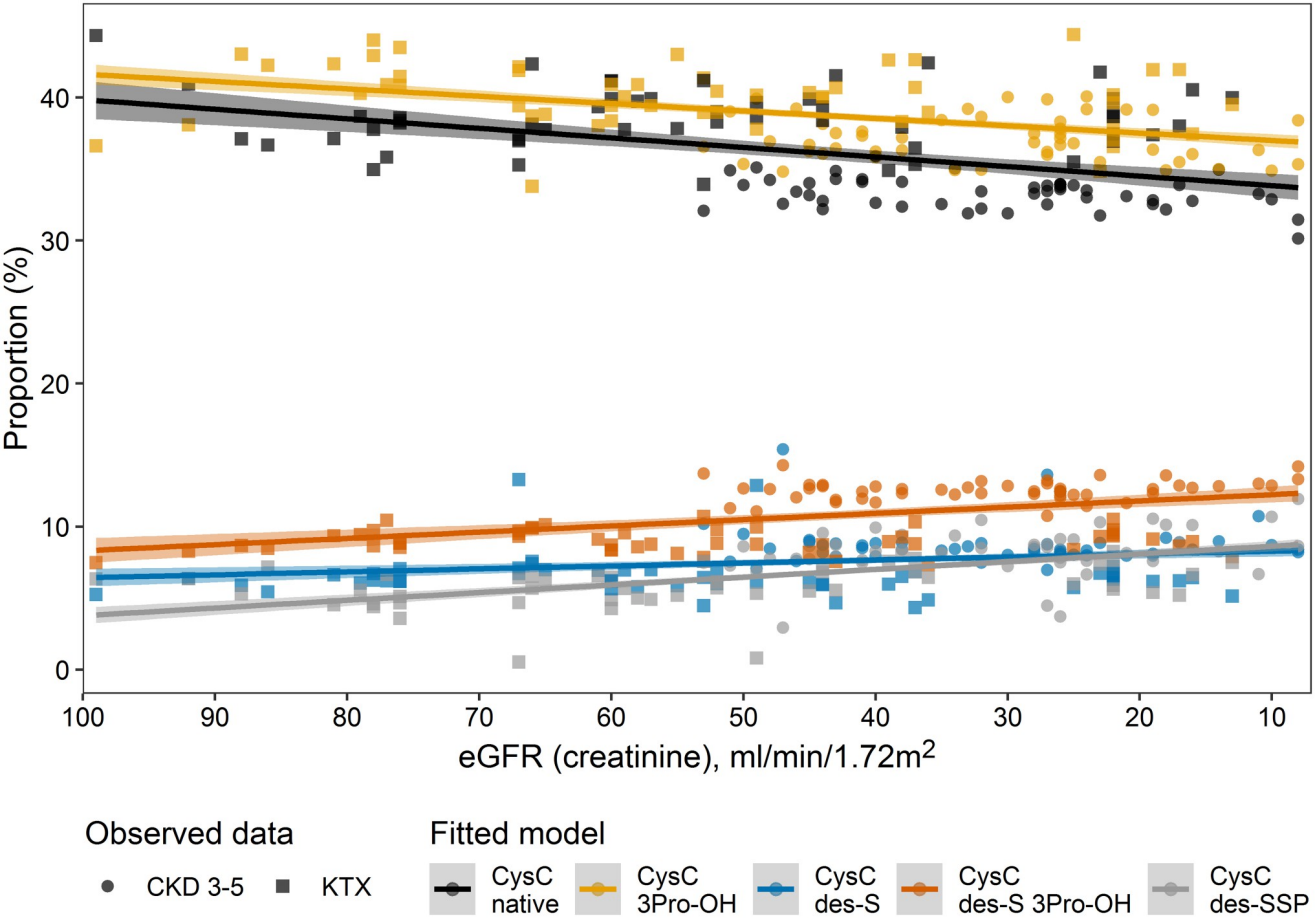
Dirichlet regression on the association of eGFR with proteoforms of cystatin C in CKD patients (n = 102, CKD 3–5 and transplant). The dots show the observed data while the lines show the fitted data. The model shows the linear estimates with adjustment for patients’ group, ATC-H02, ATC-L04 and CRP.

The observation that patients with similar eGFR but belonging to different patients groups had different proportions of proteoforms lead us to investigate the differences among the patient groups. Two candidate factors should be mentioned here, including inflammation and medication use. Inflammation was measured by plasma CRP concentrations, and revealed no major effect on the proportion of proteoforms, neither in the Dirichlet or in the beta-regression models (Fig 4, S3 Table). Even though inflammation could have been measured by other biomarkers also, the lack of association with CRP supposes that inflammation does not play a major role for the proportion of the different proteoforms.

Prescribed medicines had a rather strong association with the distribution of the different proteoforms. Indeed, immunosuppressants and especially corticosteroids (ATC-H02) were associated with a relative increase in the native forms and a relative decrease in the truncated forms. In addition, prescription of these medications explained the variance especially of the native CysC and the CysC des-S 3Pro-OH form. It has been described that corticosteroids are also associated with differences in eGFR based on CysC or creatinine in a dose dependent manner [25], a finding that is in line with our finding that larger differences between the calculated eGFR were associated with higher proportions of native CysC which were more often observed in KTX patients who also are more likely to take corticosteroids. In contrast, there are no studies investigating an association of immunosuppressants and CysC in comparison to either measured GFR or creatinine based eGFR in adults.

Other studies have also determined proteoforms of CysC. Trenchevska et al. have measured total CysC levels and four of the five proteoforms presented in this paper in a healthy population (excluding CysC des-S 3Pro-OH) [16]. Mean concentration of total CysC was around 1.0 mg/L, which was lower than in our CKD patients, and in agreement with earlier reports (26). The most abundant forms were CysC native and CysC 3Pro-OH, while concentrations of the truncated forms were lower. The distribution of the proteoforms was, however, consistent with our results, even if we did not include a healthy control group.

The same research group published a further study on patients with diabetes and CKD [17], describing the same proteoforms that we describe. Unfortunately, they did not describe the proportion of each proteoform, but only absolute values, and reported highest values of all CysC proteoforms in patients with reduced eGFR. They suggested that especially the truncated proteoforms increased independently of native CysC and could serve as a biomarker for progression of kidney disease. Our results, however, would not support this conclusion, as the proportion of proteoforms was largely independent of eGFR.

## Conclusion

In conclusion, our study showed that the concentration of proteoforms of CysC increased with decreasing renal function, however, the distribution of proteoforms was largely independent of renal function. The most important single factor that we determined that explained the distribution of proteoforms was medicine prescription. Especially prescription of corticosteroids was associated with higher native CysC levels. As this was a cross-sectional study, the clinical implications of proteoforms remain to be determined, but are, extrapolated from our findings, possibly not of crucial relevance.

## Supporting information

S1 Fig Proportion of proteoforms of CysC with estimated GFR (eGFR). Proportion of proteoforms of CysC with estimated GFR (eGFR) based on creatinine, cystatin C or both, and with total cystatin C in patients with pre-dialysis chronic kidney disease (CKD 3–5), patients receiving hemodialysis (HD) and in renal transplant recipients (KTX).
(DOCX)

S1 Table Characteristics of study population by CKD stage. (DOCX)

S2 Table Prescribed medications in the study population according to patients’ group. (DOCX)

S3 Table Association of eGFR_creat_ with % change in the proportion of CysC proteoforms in 102 patients with chronic kidney disease. Patients’ groups are CKD 3–5 and kidney transplant recipients. Estimates (95% CI) are % change in the CysC proteoform per 10 unit decrease in eGFR_creat_.
(DOCX)

## Abbreviations

CKD: Chronic kidney disease
CVD: Cardiovascular disease
CysC 3Pro-OH: 3-proline hydroxylated cystatin C
CysC des-S 3Pro-OH: N-terminal serine truncated and 3-proline hydroxylated cystatin C
CysC des-S: N-terminal serine truncated cystatin C
CysC des-SSP: N-terminal serine-serine-proline truncated cystatin C
CysC native: unmodified cystatin C
CysC: Cystatin C
eGFR: estimated glomerular filtration rate
eGFR_crea_: eGFR based on creatinine
eGFR_cysc_: eGFR based on cystatin C
