# Supplementary material for: Cystatin C proteoforms in chronic kidney disease
Source: PLoS One. 2023 Feb 1;18(2):e0269436. doi: 10.1371/journal.pone.0269436 (PMC9891521; doi:10.1371/journal.pone.0269436)
Supplement: S1 Fig — Proportion of proteoforms of CysC with estimated GFR (eGFR) based on creatinine, cystatin C or both, and with total cystatin C in patients with pre-dialysis chronic kidney disease (CKD 3–5), patients receiving hemodialysis (HD) and in renal transplant recipients (KTX). (DOCX) [file pone.0269436.s001.docx]

**S1_Figure**


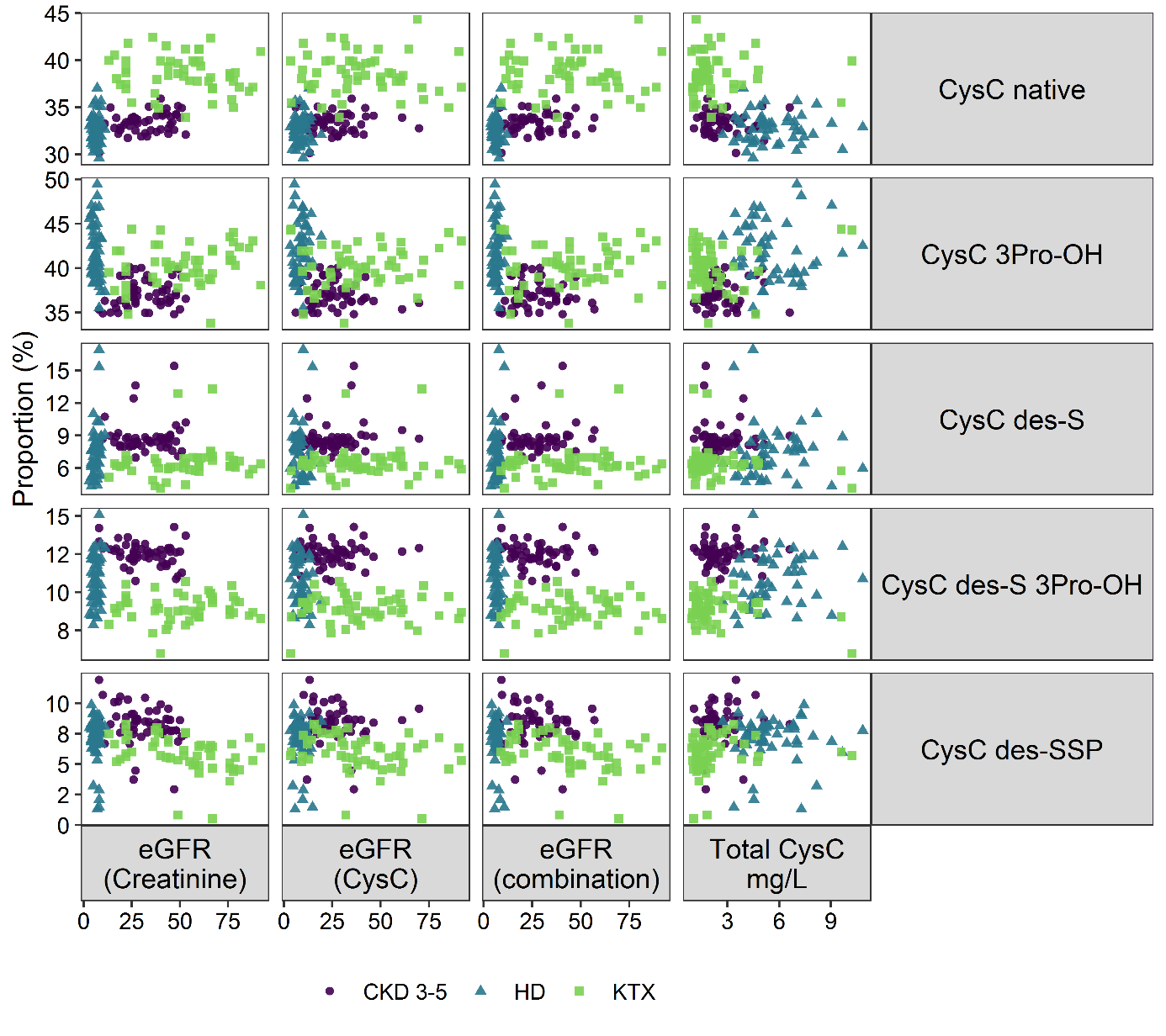


Proportion of proteoforms of CysC with estimated GFR (eGFR) based on creatinine, cystatin C or both, and with total cystatin C in patients with pre-dialysis chronic kidney disease (CKD 3-5), patients receiving hemodialysis (HD) and in renal transplant recipients (KTX).
