# Supplementary material for: Cystatin C proteoforms in chronic kidney disease
Source: PLoS One. 2023 Feb 1;18(2):e0269436. doi: 10.1371/journal.pone.0269436 (PMC9891521; doi:10.1371/journal.pone.0269436)
Supplement: S1 Table — (DOCX) [file pone.0269436.s002.docx]

| **S1_Table** | | | | | | |
| --- | --- | --- | --- | --- | --- | --- |
|  | **Total population** | **CKD stage** | | | | |
|  |  | **1-2** | **3a** | **3b** | **4** | **5** |
| n (% of total) | 137 | 22 (16) | 21 (15) | 26 (19) | 31 (23) | 37 (27) |
| Age, years | 58.62 (15.84) | 55.55 (16.47) | 57.24 (13.43) | 55.27 (15.03) | 62.61 (15.74) | 60.24 (17.15) |
| Female, n (%) | 39 (28.5 %) | 7 (31.8 %) | 4 (19 %) | 9 (34.6 %) | 11 (35.5 %) | 8 (21.6 %) |
| eGFR_crea_, ml/min/1.73m^2^ | 34.0 (23.6) | 73.9 (11.0) | 50.7 (4.2) | 38.5 (4.3) | 22.7 (3.7) | 7.1 (2.5) |
| Creatinine, µmol/L | 316 (283) | 93 (13) | 126(12) | 155 (23) | 239 (56) | 728 (218) |
| Urea, mmol/L | 15.93 (7.81) | 7.4 (1.41) | 10.12 (2.69) | 12.52 (3.04) | 19.16 (5.76) | 23.73 (6.75) |
| CRP, mg/L | 5.1 (12.9) | 2.8 (3.1) | 3.3 (4.5) | 3.0 (3.9) | 4.0 (4.0) | 8.2 (24.0) |
| Cystatin C, ug/mL | 3.25 (2.12) | 1.31 (0.31) | 1.9 (0.8) | 2.37 (1.65) | 3.4 (1.44) | 5.67 (1.74) |
| BMI, kg/m^2^ | 26.38 (4.8) | 26 (4.23) | 28.49 (6.12) | 27.26 (4.64) | 26.63 (4.67) | 24.58 (4) |
| Continuous variables presented as means (SD) and categorical variables reported as counts (%). eGFR based on equations for creatinine by CKD-EPI (6) .  BMI, body mass index; CKD 3-5, pre-dialysis chronic kidney disease stage 3-5; CysC, Cystatin C; CysC native, unmodified CysC; CysC 3, Pro-OH: 3-proline hydroxylated CysC; CysC des-S, N-terminal serine truncated CysC; CysC des-S 3Pro-OH, N-terminal truncated serine and 3-proline hydroxylated CysC; CysC des-SSP, N-terminal serine-serine-proline truncated CysC; eGFR, estimated glomerular filtration rate; HD, end-stage renal disease hemodialysis; KTX, renal transplant recipient. ATC-H02 – corticosteroids, ATC-L04 - immunosuppressants | | | | | | |
