# Supplementary material for: Cystatin C proteoforms in chronic kidney disease
Source: PLoS One. 2023 Feb 1;18(2):e0269436. doi: 10.1371/journal.pone.0269436 (PMC9891521; doi:10.1371/journal.pone.0269436)
Supplement: S2 Table — (DOCX) [file pone.0269436.s003.docx]

| **S2_Table** | | | | |
| --- | --- | --- | --- | --- |
| **ATC Classification** | | **CKD 5-3** | **HD** | **KTX** |
| **A** | **Alimmentary tract and metabolism** | **42 (79.2)** | **51 (100.0)** | **46 (86.8)** |
| A01 | Stomatological preparations | 0 (0.0) | 0 (0.0) | 0 (0.0) |
| A02 | Drugs for acid related disorders | 26 (49.1) | 39 (76.5) | 31 (58.5) |
| A03 | Drugs for functional gastrointestinal disorders | 1 (1.9) | 2 (3.9) | 0 (0.0) |
| A04 | Antiemetics and antinauseants | 0 (0.0) | 2 (3.9) | 0 (0.0) |
| A06 | Drugs for constipation | 1 (1.9) | 10 (19.6) | 0 (0.0) |
| A07 | Antidiarrheals, intestinal antiinflammatory/antiinfective agents | 1 (1.9) | 1 (2.0) | 2 (3.8) |
| A10 | Drugs used in diabetes | 12 (22.6) | 13 (25.5) | 7 (13.2) |
| A11 | Vitamins | 26 (49.1) | 48 (94.1) | 11 (20.8) |
| A12 | Mineral supplements | 5 (9.4) | 31 (60.8) | 38 (71.7) |
| **B** | **Blood and blood forming organs** | **29 (54.7)** | **51 (100.0)** | **27 (50.9)** |
| B01 | Antithrombotic agents | 24 (45) | 49 (96.1) | 25 (47.2) |
| B03 | Antianemic preparations | 6 (11.3) | 47 (92.2) | 5 (9.4) |
| **C** | **Cardiovascular system** | **48 (90.6)** | **49 (96.1)** | **48 (90.6)** |
| C01 | Cardiac therapy | 2 (3.8) | 5 (9.8) | 1 (1.9) |
| C02 | Antihypertensives | 5 (9.4) | 6 (11.8) | 0 (0.0) |
| C03 | Diuretics | 11 (20.8) | 30 (58.8) | 13 (24.5) |
| C07 | Beta blocking agents | 22 (41.5) | 39 (76.5) | 23 (43.4) |
| C08 | Calcium channel blockers | 18 (34.0) | 35 (68.6) | 23 (43.4) |
| C09 | Agents acting on the renin-angiotensin system | 37 (69.8) | 22 (43.1) | 29 (54.7) |
| C10 | Lipid modifying agents | 29 (54.7) | 31 (60.8) | 38 (71.7) |
| **D** | **Dermatologicals** | **0 (0.0)** | **2 (3.9)** | **2 (3.8)** |
| D01 | Antifungals for dermatological use | 0 (0.0) | 0 (0.0) | 1 (1.9) |
| D05 | Antipsoriatics | 0 (0.0) | 0 (0.0) | 1 (1.9) |
| D07 | Corticosteroids, dermatological preparations | 0 (0.0) | 2 (3.9) | 0 (0.0) |
| **G** | **Genito urinary system and sex hormones** | **5 (9.4)** | **8 (15.7)** | **5 (9.4)** |
| G03 | Sex hormones and modulators of the genital system | 1 (1.9) | 0 (0.0) | 1 (1.9) |
| G04 | Urologicals | 4 (7.5) | 8 (15.7) | 4 (7.5) |
| **H** | **Systemic hormonal preparations, excluding sex hormones and insulins** | **9 (17.0)** | **23 (45.1)** | **52 (98.1)** |
| H02 | Corticosteroids for systemic use | 5 (9.4) | 9 (17.6) | 51 (96.2) |
| H03 | Thyroid therapy | 3 (5.7) | 5 (9.8) | 1 (1.9) |
| H05 | Calcium homeostasis | 1 (1.9) | 11 (21.6) | 0 (0.) |
| **J** | **Antiinfective for systemic use** | **0 (0.0)** | **3 (5.9)** | **5 (9.4)** |
| J01 | Antibacterials for systemic use | 0 (0.0) | 2 (3.9) | 4 (7.5) |
| J02 | Antimycotics for systemic use | 0 (0.0) | 1 (2.0) | 0 (0.0) |
| J05 | Antivirals for systemic use | 0 (0.0) | 0 (0.0) | 1 (1.9) |
| **L** | **Antineoplastic and immunomodulating agents** | **3 (5.7)** | **9 (17.6)** | **53 (100.0)** |
| L02 | Endocrine therapy | 0 (0.0) | 2 (3.9) | 0 (0.0) |
| L04 | Immunosuppressants | 3 (5.7) | 7 (13.7) | 53 (100.0) |
| **M** | **Musculo-skeletal system** | **16 (30.2)** | **12 (23.5)** | **19 (35.8)** |
| M01 | Antiinflammatory and antirheumatic products | 0 (0.0) | 0 (0.0) | 0 (0.0) |
| M04 | Antigout preparations | 14 (26.4) | 12 (23.5) | 11 (20.8) |
| M05 | Drugs for treatment of bone diseases | 2 (3.8) | 0 (0.0) | 8 (15.1) |
| **N** | **Nervous system** | **12 (22.6)** | **29 (56.9)** | **9 (17.0)** |
| N02 | Analgesics | 6 (11.3) | 8 (15.7) | 2 (3.8) |
| N03 | Antiepileptics | 2 (3.8) | 7 (13.7) | 1 (1.9) |
| N04 | Anti-parkinson drugs | 2 (3.8) | 3 (5.9) | 0 (0.0) |
| N05 | Psycholeptics | 9 (17.0) | 24 (47.1) | 4 (7.5) |
| N06 | Psychoanaleptics | 6 (11.3) | 4 (7.8) | 4 (7.5) |
| N07 | Other nervous system drugs | 0 (0.0) | 2 (3.9) | 0 (0.0) |
| **P** | **Antiparasitic products, insecticides and repellents** | **0 (0.0)** | **0 (0.0)** | **3 (5.7)** |
| P01 | Antiprotozoals | 0 (0.0) | 0 (0.0) | 3 (5.7) |
| **R** | **Respiratory system** | **6 (11.3)** | **7 (13.7)** | **4 (7.5)** |
| R01 | Nasal preparations | 1 (1.9) | 0 (0.0) | 1 (1.9) |
| R03 | Drugs for obstructive airway diseases | 3 (5.7) | 5 (9.8) | 1 (1.9) |
| R05 | Cough and cold preparations | 0 (0.0) | 1 (2.0) | 0 (0.0) |
| R06 | Antihistamines for systemic use | 3 (5.7) | 2 (3.9) | 2 (3.8) |
| **S** | **Sensory organs** | **1 (1.9)** | **0 (0.0)** | **2 (3.8)** |
| S01 | Ophthalmologicals | 1 (1.9) | 0 (0.0) | 2 (3.8) |
| **V** | **Various** | **4 (7.5)** | **47 (92.2)** | **0 (0.0)** |
| V03 | All other therapeutic products | 4 (7.5) | 47 (92.2) | 0 (0.0) |
| Number and percentage of patients receiving at least one prescribed mediation from this group; ATC first level, and ATC second level.  ATC, anatomical therapeutic chemical; CKD 3-5, pre-dialysis patients with chronic kidney disease stage 3-5; HD, hemodialysis patients; KTX, renal transplant recipients. | | | | |
