# Supplementary material for: Cystatin C proteoforms in chronic kidney disease
Source: PLoS One. 2023 Feb 1;18(2):e0269436. doi: 10.1371/journal.pone.0269436 (PMC9891521; doi:10.1371/journal.pone.0269436)
Supplement: S3 Table — Patients’ groups are CKD 3–5 and kidney transplant recipients. Estimates (95% CI) are % change in the CysC proteoform per 10 unit decrease in eGFRcreat. (DOCX) [file pone.0269436.s004.docx]

| S3_Table | | | | | |
| --- | --- | --- | --- | --- | --- |
| Model* | CysC native | CysC 3Pro-OH | CysC des-S | CysC des-S 3Pro-OH | CysC deS-SSP |
| Model1 | -3.0 (-4.0, -1.9) | -2.1 (-2.9, -1.2) | 3.5 (1.1, 5.8) | 4.9 (3.1, 6.7) | 8.8 (5.6, 12.1) |
| Model2 | -0.3 (-1.0, 0.5) | -0.7 (-1.5, 0.2) | -0.9 (-2.9, 1.2) | 0.4 (-0.5, 1.4) | 5.0 (1.7, 8.4) |
| Model3 | -0.5 (-1.4, 0.4) | -1.1 (-2.0, -0.2) | -0.0 (-2.4, 2.3) | 1.0 (-0.4, 2.5) | 5.4 (2.0, 9.0) |
| Model4 | -0.5 (-1.4, 0.3) | -0.8 (-1.7, 0.0) | -0.5 (-2.6, 1.7) | 0.9 (-0.3, 2.1) | 5.4 (2.1, 8.8) |
| Model5 | -3.0 (-4.0, -1.9) | 2.2 (-3.0, -1.3) | 3.4 (1.1, 5.8) | 4.9 (3.1, 6.6) | 9.2 (6.0, 12.5) |
| Model6 | -0.2 (-1.0, 0.6) | -1.0 (-1.8, -0.1 | -0.5 (-2.6, 1.6) | 0.5 (-0.4, 1.4) | 5.3 (2.0, 8.7 |
| * Model 1: unadjusted, Model 2: adjusted for patients’ group, Model 3: adjusted for ATC-H02 (hormones), Model 4: adjusted for ATC-L04 (immunosuppressants), Model 5: adjusted for CRP, Model 6: adjusted for patients group, ATC-H02, ATC-L04, CRP  CysC, cystatin C; CysC native, unmodified CysC; CysC 3Pro-OH: 3-proline hydroxylated CysC; CysC des-S, n-terminal serine truncated CysC; CysC des-S 3pro-oh, n-terminal truncated serine and 3-proline hydroxylated CysC; CysC des-SSP, n-terminal serine-serine-proline truncated CysC; | | | | | |
